# Ischemia/Reperfusion-Induced Translocation of PKCβII to Mitochondria as an Important Mediator of a Protective Signaling Mechanism in an Ischemia-Resistant Region of the Hippocampus

**DOI:** 10.1007/s11064-017-2263-3

**Published:** 2017-04-12

**Authors:** Olga Krupska, Anna Sarnowska, Bartlomiej Fedorczyk, Magdalena Gewartowska, Aleksandra Misicka, Barbara Zablocka, Malgorzata Beresewicz

**Affiliations:** 1 Molecular Biology Unit, Mossakowski Medical Research Centre, PAS, Warsaw, Poland; 2 Stem Cell Bioengineering Unit, Mossakowski Medical Research Centre, PAS, Warsaw, Poland; 3 Faculty of Chemistry, University of Warsaw, Warsaw, Poland; 4 Electron Microscopy Platform, Mossakowski Medical Research Centre, PAS, Warsaw, Poland; 5 Department of Neuropeptides, Mossakowski Medical Research Centre, PAS, Warsaw, Poland

**Keywords:** Cerebral ischemia, Protein kinase C, PKCβII, Endogenous neuroprotection, Mitochondria

## Abstract

Emerging reports indicate that activated PKC isoforms that translocate to the mitochondria are pro- or anti-apoptotic to mitochondrial function. Here, we concentrate on the role of PKCβ translocated to mitochondria in relation to the fate of neurons following cerebral ischemia. As we have demonstrated previously ischemia/reperfusion injury (I/R) results in translocation of PKCβ from cytoplasm to mitochondria, but only in ischemia-resistant regions of the hippocampus (CA2-4, DG), we hypothesize that this translocation may be a mediator of a protective signaling mechanism in this region. We have therefore sought to demonstrate a possible relationship between PKCβII translocation and ischemic resistance of CA2-4, DG. Here, we reveal that I/R injury induces a marked elevation of PKCβII protein levels, and consequent enzymatic activity, in CA2-4, DG in the mitochondrial fraction. Moreover, the administration of an isozyme-selective PKCβII inhibitor showed inhibition of I/R-induced translocation of PKCβII to the mitochondria and an increase in neuronal death following I/R injury in CA1 and CA2-4, DG in both an in vivo and an in vitro model of ischemia. The present results suggest that PKCβII translocated to mitochondria is involved in providing ischemic resistance of CA2-4, DG. However, the exact mechanisms by which PKCβII-mediated neuroprotection is achieved are in need of further elucidation.

## Introduction

As mitochondria are the principal mediators of the cell death that occurs during cerebral ischemia, they are important targets for neuroprotective interventions. Recent reports indicate participation of the protein kinase C (PKC) family of serine/threonine kinases in the regulation of mitochondrial metabolism capable of determining the fate of cells following ischemic stress. Activation of multiple PKC isozymes has been shown to occur after ischemia/reperfusion (I/R) injury, in multiple organs including the heart, liver, kidney and brain, suggesting its involvement in ischemic response pathways [1]. As activity increases, simultaneous translocation of PKCs to specialized membrane compartments including mitochondria is observed. However, the role of individual isozymes of PKCs in ischemic injury appears rather contradictory, given that these can mediate different and sometimes opposing functions following activation by the same stimulus. For example, two isozymes of a novel type of PKC, PKCε and PKCδ, show opposite effects on apoptosis [2].

PKCε appears to play a critical role in conferring protection against neuronal ischemia/reperfusion [3]. Since activation of PKCε leads to its translocation to mitochondria, the suggested role is in the promotion of cell survival through regulation of mitochondrial function and signaling [4–6]. The further hypothesis relates to responsibility for ischemic tolerance. PKCε can regulate key cytoprotective mitochondrial functions, including electron transport chain activity, the generation of reactive oxygen species (ROS), mitochondrial permeability transition, and detoxification of reactive aldehydes [7–11].

Like PKCε, PKCδ also undergoes post-I/R injury translocation to the mitochondria, where it affects its metabolism. However, a difference in this case is that PKCδ has an active involvement in cell apoptosis. In circumstances of cardiac ischemia, translocation of PKCδ to the mitochondria during reperfusion likely results in superoxide anion production and a loss of mitochondrial function [12], activation of pyruvate dehydrogenase kinase 2 and phosphorylation-dependent inhibition of the mitochondrial enzyme pyruvate dehydrogenase [13], and inhibition of F_1_F_0_ activity via an interaction with the δ subunit of F_1_F_0_-ATP synthase [14].

There are also data reporting the role of other PKCs in regulating mitochondrial function and signaling, including with reference to PKCα [15] and PKCβ [16, 17].

In turn, the work described here has concentrated on the role of PKCβ in mitochondria in the context of I/R injury in the brain. To study this aspect, we used a well-known animal model of cerebral ischemia in the gerbil in which 5 min of bilateral ligation of common carotid arteries results in selective, delayed death of pyramidal cells in the hippocampal CA1 region, while the adjacent CA2-4, DG regions remain relatively resistant. CA2-4, DG therefore appear to serve as a good model for study of mechanisms underlying survival in hippocampal neurons.

Using this gerbil model, we have previously demonstrated that, following I/R injury, PKCβ translocates to the mitochondrial fraction, mainly in CA2-4, DG (ischemia-resistant), but not in CA1 (the ischemia-vulnerable part of the hippocampus) [17]. In this context, we hypothesize that the translocation may mediate a protective signaling mechanism in CA2-4, DG, following I/R injury. This paper therefore describes a series of experiments performed to determine possible relationships between PKCβ translocation and ischemic resistance of the hippocampal CA2-4, DG area.

The two PKCβ isozymes are βI and βII, which are differentially spliced products of the same gene. The two isozymes display 100% identity over their first 621 amino acids, but then diverge in the last 50–52 carboxyl-terminal residues. Importantly, these divergences are 100% conserved across rat, rabbit, and human suggesting distinct functions encoded by the two gene products [18]. Additionally, isozyme-specific differences in the subcellular localization and function of PKCβI and βII have been reported previously. Using immunofluorescence, several groups have observed an isozyme-specific association of PKCβII with mitochondria and the actin cytoskeleton [19, 20].

Here, we reveal that among the PKCβ isozymes, it is PKCβII rather than βI whose levels increases in mitochondria in the ischemia-resistant part of the hippocampus following I/R injury. An in vitro PKCβII activity assay shows that PKCβII translocated to mitochondria has enzymatic activity. Moreover, we used an isozyme-selective PKCβII inhibitor (βIIV5-3), which was previously developed in the Daria Mochly-Rosen lab [21, 22]. This six-amino-acid peptide (corresponding to amino acids 645–650 (QEVIRN) of the PKCβII V5 region) was derived from the interaction site between PKCβII and its receptor for activated C kinase (RACK1). It prevents interaction between PKCβII and RACK1, leading to a selective loss of function mediated by PKCβII, without the function of other PKC isozymes being affected. Here, we show that the isozyme-selective PKCβII inhibitor decreases I/R-induced translocation of PKCβII to the mitochondrial fraction, and increases neuronal death following I/R injury in both the in vitro and the in vivo models of ischemia. The results presented here thus suggest the involvement of PKCβII in the ischemic resistance displayed by CA2-4, DG.

## Materials and Methods

### Ethical Statement and Animals

All experimental procedures were approved by the Local Ethics Committee for Animal Experimentation and every effort was made to minimize animal suffering. Wistar rats (7-day-old pups) and Mongolian gerbils (*Meriones unguiculatus*) were obtained from the Animal House of the Mossakowski Medical Research Centre of the Polish Academy of Sciences.

### Transient Brain Ischemia in Gerbils

Gerbils weighing 50–70 g were subjected to transient brain ischemia by means of a 5-min bilateral ligation of common carotid arteries under isoflurane anesthesia, in strictly controlled normothermic conditions as described previously [23]. Following ischemia, the animals recovered for 1 or 96 h prior to decapitation, with the CA1 and CA2-4, DG regions of the hippocampus then being isolated for mitochondrial fraction isolation. Hippocampi from non-treated animals served as controls.

βIIV5-3 peptide was used to establish the effect of PKCβII inhibition on PKCβII immunoreactivity in mitochondria and neuronal death. This peptide is an isozyme-selective inhibitor of PKCβII developed by the Daria Mochly-Rosen lab and kindly offered to us [21, 22]. In our study, βIIV5-3 peptide was dissolved in saline and injected directly to the left carotid artery in the course of the ischemic insult in the gerbil model (3 mg/kg). The animals were allowed to recover for 1 h and isolated hippocampi were used for mitochondrial fraction isolation or for 7 days for histological purposes. The applied dose of βIIV5-3 peptide was selected on the basis of reports in the literature [22, 24, 25].

### TAT Peptide Synthesis

βIIV5-3 peptide is conjugated to the carrier peptide TAT via a reducible Cys–Cys disulfide bond that allows for release of βIIV5-3 following uptake. TAT peptide (YGRKKRRQRRR) linked with Cys residue at the N-terminus (H-Cys-TAT) was therefore produced. The desired peptide was synthesized manually by means of Solid Phase Peptide Synthesis via the Fmoc/tBu strategy on Wang resin. After synthesis, the peptide was cleaved from the resin, and a crude product purified by RP-HPLC. Structure was confirmed by ESI-MS: calc. [M + 4H]4 + 416.4977 found 416,4959.

### Sample Preparation and Isolation of Pure Mitochondria

A particulate and soluble fraction together with a pure-mitochondria fraction were obtained from the CA1 and CA2-4, DG regions of the hippocampi of control and ischemic gerbils. Hippocampi were homogenized in ice-cold isotonic buffer (15 mM Tris/HCl, pH 7.5, 0.25 M sucrose, 1 mM MgCl_2_, 1 mM EGTA, 2 mM EDTA, 1 mM PMSF and 1 mM DTT), prior to centrifugation (1000*g*, 10 min, 4 °C). The supernatant was centrifuged at 11,000*g* for 20 min at 4 °C to yield a particulate fraction enriched with mitochondria (P2), as well as a soluble fraction (S2). The pure mitochondrial pellet was obtained after centrifugation of P2 (100,000*g*, 30 min, 4 °C) with 12% Ficoll, as described earlier [26]. The protein concentration was determined using a Modified Lowry Protein Assay Kit (Thermo Scientific). Purity of the mitochondrial fraction was confirmed by western blot analysis using antibodies directed against plasma membrane (cadherin, Abcam), cytosol (lactate dehydrogenase, Proteintech Group) endoplasmic reticulum (inositol trisphosphate receptor, Affinity Bioreagents) and mitochondrial proteins (voltage-dependent anion channel, Santa Cruz Biotechnology; cytochrome c oxidase, Invitrogen)—data not shown.

### Western Blot Analysis

The mitochondrial fraction or the particulate and soluble fractions (20 µg) were separated by 10% SDS-PAGE, transferred to nitrocellulose membrane (Amersham Protran, GE Healthcare) and analysed by western blot using mouse monoclonal anti-PKCβ (BD Transduction Laboratories), as well as rabbit polyclonal anti-PKCβI and βII antibodies (Abcam). Equal protein loading was confirmed by cox IV levels (mouse monoclonal anti-cytochrome c oxidase, Invitrogen). Protein bands were detected with horseradish peroxidase-coupled secondary antibody (rabbit anti-mouse and goat anti-rabbit), and enhanced with chemiluminescent substrate (Amersham ECL Western Blotting Detection Reagents, GE Healthcare). The bands were evaluated by densitometry and normalized.

### In Vitro PKCβII Activity Assay

Pure mitochondria (100 µg) from the CA2-4, DG region of control and ischemic gerbils (I/R 1 h and I/R 96 h) were incubated in lysis buffer (20 mM Tris HCl pH 7.5, 1% Triton, 0.5% NP-40, 0.1 mM CaCl_2_, 1 mM PMSF, phosphatase and protease inhibitor cocktail (Sigma)) for 30 min at 4 °C and centrifuged (12,000*g*, 15 min, 4 °C). The supernatants were incubated (2 h, RT) with anti-PKCβII antibody (Abcam) attached to magnetic Dynabeads Protein G (Novex, Life Technologies) to achieve purification of endogenous PKCβII from the mitochondrial fraction. After extensive washing, the beads conjugated to PKCβII were incubated with 200 µM ATP (Cell Signaling) and 0.1 mg/ml Histone H1 (Millipore) for 30 min at 37 °C. The reaction mixture was then transferred to another tube and boiled in 5× SDS protein sample buffer (5 min, 100 °C). Samples were separated by SDS-PAGE and analyzed with anti-phospho-Histone H1 (Upstate) and anti-Histone H1 (Abcam) antibodies.

### Immunogold Electron Microscopy

Immunolocalization of PKCβII in mitochondria was verified by immunogold electron microscopy. Gerbils from the control group, as well as those categorized as ischemic (I/R 1 h + saline) and ischemic treated with βIIV5-3 peptide (I/R 1 h + βIIV5-3) were perfused with ice-cold 4% paraformaldehyde just 1 h after reperfusion. Isolated hippocampi were divided into the CA1 and CA2-4, DG regions, prior to post-fixing overnight in 4% paraformaldehyde. Tissues were next treated with 1% osmium tetroxide for 1 h, dehydrated in an ethanol gradient, and finally embedded in Epon. Sections were cut using an LKB-NOVA ultramicrotome, and collected on nickel grids covered with formvar film, incubated in 10% hydrogen peroxide for 10 min and rinsed in PBST, the sections were incubated with blocking solution (0.1% BSA), before being incubated with anti-mouse PKCβII antibody (Santa Cruz, 1:30 in PBS), overnight at 4 °C. Immunodetection was achieved using secondary anti-mouse (1:50) antibody conjugated to 12 nm gold particles (Jackson ImmunoResearch). Examination of all sections was carried out using a JEM 1011 electron microscope at 100 kV. Incubation with secondary antibody (IgG) alone represented a control.

### Translocation of PKCβII

To establish whether changes in PKCβII immunoreactivity are due to its translocation to mitochondria following I/R injury, hippocampi from the control, as well as the groups termed ischemic (I/R 1 h + saline) and ischemic treated with βIIV5-3 peptide, were used in the isolation of the particulate (P2) and soluble (S2) fractions (obtained from the CA2-4, DG regions of hippocampi). Obtained fractions were separated by SDS-PAGE and analyzed by western blot with anti-PKCβII antibody (Abcam). Equal protein loading was confirmed by GAPDH level (mouse monoclonal anti-GAPDH, Santa Cruz).

### Organotypic Hippocampal Slice Culture

Organotypic hippocampal slices were used to examine the effect of PKCβII inhibition on neuronal death. The slices were prepared from 7-day old Wistar rats, after Stoppini, though with slight modifications [27]. Four hundred micrometer-thick slices were cultured in medium containing 50% Neurobasal (Gibco), 22% HBSS, 25% horse serum (Gibco), 1 M HEPES, 5 mg/ml glucose and 0.8% Antibiotic Antimycotic Solution (Sigma). Cultures were started in a 25% horse serum-containing medium which was gradually replaced (from DIV three through to eight) by a serum-free medium, which contained additionally 1:50 B27 supplement (Gibco). Cultures were maintained in a humidified atmosphere of air and 5% CO_2_, at 36 °C for 9 days. Neuronal death was induced by *N*-methyl-d-aspartic acid (NMDA) (100 µM, 3 h in culture), which was added to the culture simultaneously with various (0.5, 1, 2 µM) concentrations of βIIV5-3 peptide. After 3 h of incubation, NMDA was removed while βIIV5-3 peptide was present in the culture medium until the end of the experiment. Quantification of cell death was achieved by reference to the intensity of fluorescent cell death marker–propidium iodide (PI) from each slice. Obtained values were normalized to maximal fluorescent intensity, obtained by treating cells with 500 µM NMDA.

### Hematoxylin and Eosin Staining and TUNEL Assay

Hematoxylin and eosin staining (H/E) and terminal deoxynucleotidyl transferase dUTP nick end labelling (TUNEL; In situ Cell Death Detection Kit, Roche) were used to analyze the influence of βIIV5-3 on neuronal cell death after I/R injury. Gerbils from the control, ischemic (I/R + saline) and ischemic subjected to βIIV5-3 (I/R + βIIV5-3) groups were treated as described above. After 7 days of recovery animals were perfused with ice-cold 4% paraformaldehyde in PBS under ketamine/xylazine anesthesia. Their brains were then paraffin-embedded, fixed and cut into 10 µm-thick sections. The morphology of brain slices was investigated following standard H/E staining or TUNEL assay performed in line with directions from the manufacturer (Roche). The extent of cell damage in the CA1 hippocampal region was quantified as the mean number of remaining intact neurons (H/E staining). Intact neurons were counted from three well-defined sections of 200 µm length within the CA1 region, using an image analysis system (Zeiss Zen Microscopy Software). Quantification of TUNEL staining was based on fluorescent intensity measurements of each slice and normalization to a maximal fluorescent value obtained by treating slices with DNase (3000U).

### Statistics

The significance of differences among groups was calculated using one-way analysis of variance followed by Tukey’s Multiple Comparison Test. A value of p < 0.05 was considered significant.

## Results

### PKCβ Immunoreactivity in the Mitochondrial Fraction

**Fig. 1:**
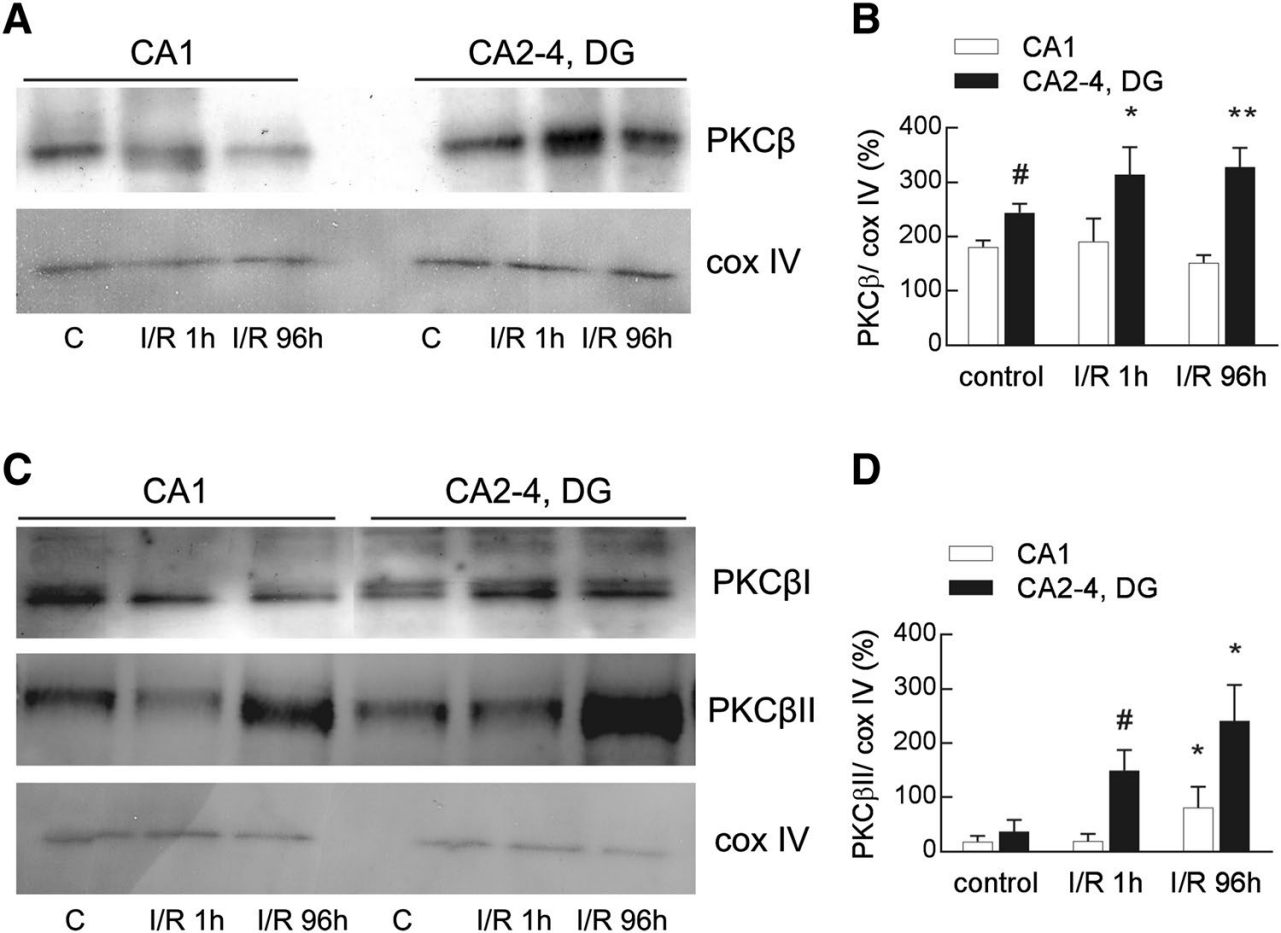
Ischemia/reperfusion-induced changes in total PKCβ and PKCβII immunoreactivity in the mitochondrial fraction isolated from the ischemia-vulnerable (CA1) and ischemia-resistant (CA2-4, DG) parts of the hippocampus. Hippocampi were obtained from control and ischemic animals subjected to 5 min ischemia and 1 or 96 h of reperfusion (I/R 1 h and I/R 96 h). The pure mitochondrial fractions (20 µg) were separated by 10% SDS-PAGE and analyzed by western blot with anti-PKCβ or anti-PKCβI and βII and anti-cox IV to assess the gel loading. **a** The immunoblot showing total PKCβ immunoreactivity in the mitochondrial fraction is representative of five independent experiments. **b** Densities of PKCβ bands were evaluated and data are expressed as a percentage of cox IV (mean ± SD, n = 5). *p < 0.05, **p < 0.01 versus control CA2-4, DG, ^#^p < 0.05 versus control CA1. **c** The representative immunoblots of four independent experiments show PKCβI and βII immunoreactivity in the mitochondrial fraction. **d** Densities of PKCβII bands were evaluated and data are expressed as a percentage of cox IV (mean ± SD, n = 4). *p < 0.05 versus I/R 1 h, ^#^p < 0.01 versus control

As we have shown previously, I/R injury induces a significant elevation of PKCβ immunoreactivity in the mitochondrial fraction, but mainly in the ischemia-resistant part of the hippocampus (CA2-4, DG) [17]. The increase appears as soon as 1 h after I/R injury, and persists even up to 96 h after the event (Fig. 1a, b). Western blots identified specific PKCβ isozymes, and revealed that it is rather PKCβII than PKCβI which increases in the mitochondrial fraction after I/R (Fig. 1c).

PKCβII immunoreactivity showed a significant increase 1 and 96 h after I/R but mainly in CA2-4, DG. In contrast, a slight increase of its immunoreactivity was observed in the CA1 region, but only 96 h after I/R (Fig. 1c, d). PKCβI was identified in the mitochondria, but its immunoreactivity did not change with I/R injury in any region of the hippocampus (Fig. 1c, densitometry measurements not shown).

### Enzymatic Activity of PKCβII in Mitochondrial Fraction

**Fig. 2:**
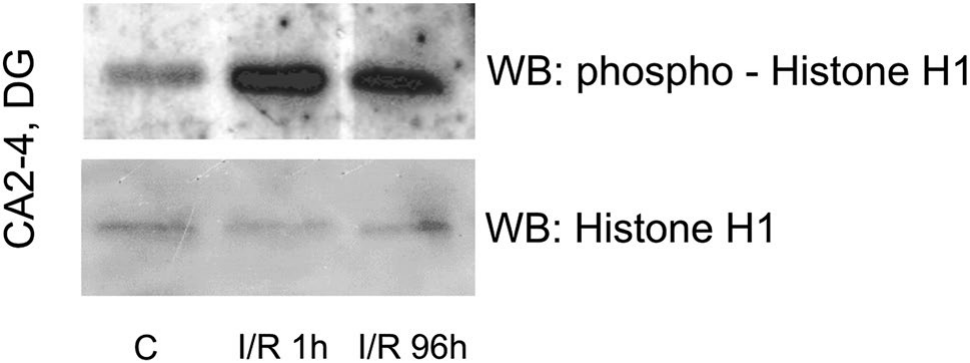
Postischemic enzymatic activity of PKCβII in mitochondrial fraction. PKCβII-immunoprecipitated samples obtained from mitochondrial fraction from the CA2-4/DG of control and ischemic animals (I/R 1 h and I/R 96 h) were incubated with ATP and Histone H1 as a substrate. The reaction mixture was separated by SDS-PAGE and analyzed with anti-phospho-Histone H1 and anti-Histone H1 antibody. The immunoblot shown represents two independent experiments

An in vitro PKCβII activity assay was performed to assess enzymatic activity of PKCβII in the mitochondrial fraction. PKCβII-immunoprecipitated samples obtained from the mitochondrial fraction from the CA2-4, DG regions of control and ischemic animals (I/R 1 h and I/R 96 h) were incubated with ATP and Histone H1 as a substrate. The reaction mixture was separated by SDS-PAGE and analysed with anti-phospho-Histone H1 and anti-Histone H1 antibody. As Fig. 2 shows, both 1 and 96 h after I/R the phosphorylated form of Histone H1 immunoreactivity is elevated, as accompanied by a lack of change in Histone H1 immunoreactivity. These results indicate that PKCβII translocated to mitochondria is active enzymatically.

### Effect of βIIV5-3 Peptide on PKCβII Translocation to the Mitochondrial Fraction

**Fig. 3:**
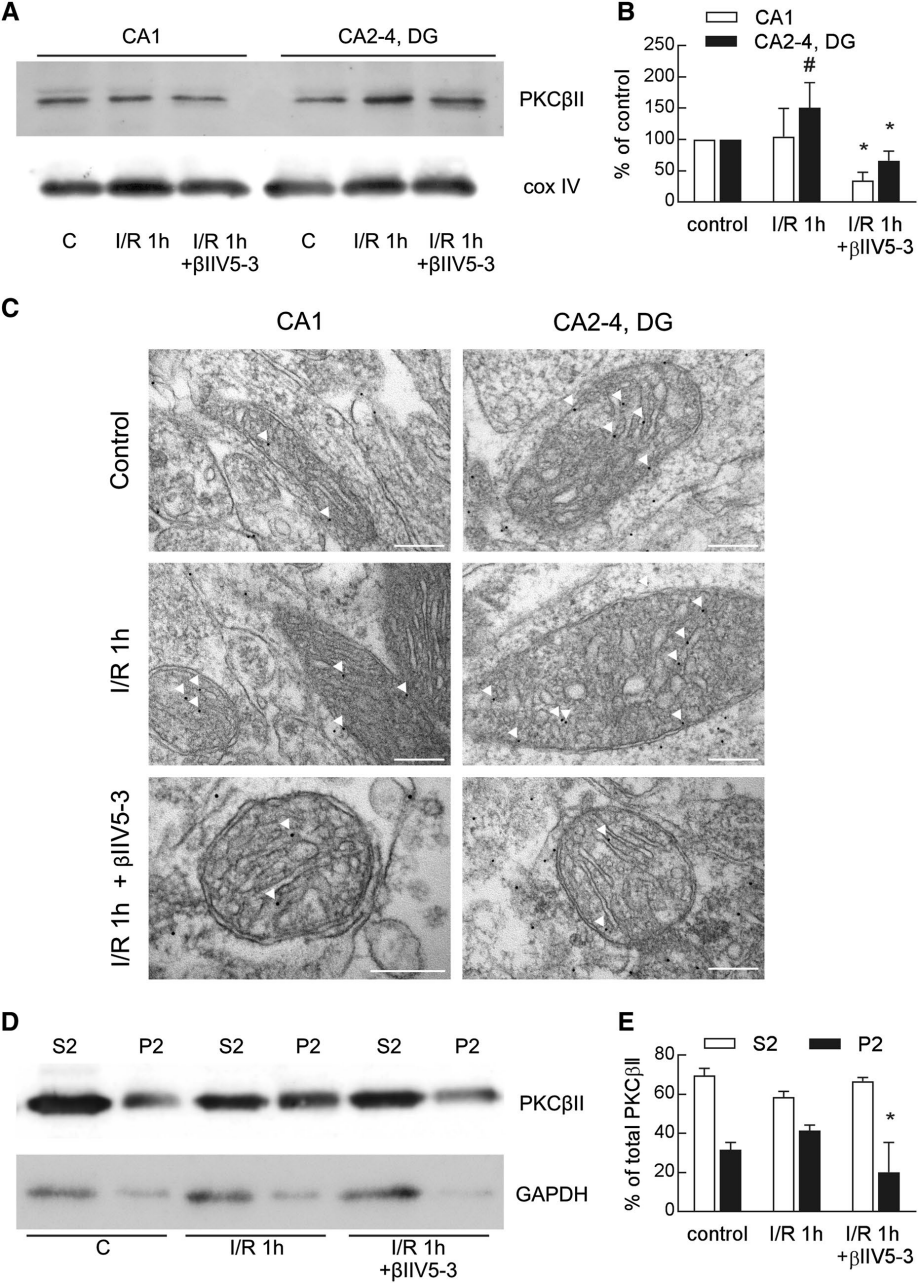
Effect of the PKCβII isozyme-selective inhibitor on PKCβII translocation to the mitochondrial fraction. The PKCβII isozyme-selective inhibitor (βIIV5-3) or saline were applied during the ischemic procedure to the left carotid artery. One hour after ischemia/reperfusion (I/R) the mitochondrial fraction from the CA1 and CA2-4, DG regions of hippocampi or the soluble and particulate (mitochondria-enriched) fraction from the CA2-4, DG region were obtained. **a** Twenty microgram of pure mitochondrial fractions were separated by 10% SDS-PAGE and analyzed by western blot with anti-PKCβII and anti-cox IV to assess the gel loading. The immunoblot is representative of four independent experiments. **b** Densities of PKCβII bands were evaluated and data are expressed as a percentage of values in the controls (mean ± SD, n = 4). *p < 0.01 versus I/R, ^#^p < 0.05 versus control. **c** Electron micrographs of PKCβII immunogold labelling in mitochondria. Mitochondrial localization is indicated by arrows. *Scale bar* 200 nm. **d** Thirty microgram of soluble (S2) and particulate fraction (P2) were separated by 10% SDS-PAGE and analyzed by western blot with anti-PKCβII and anti-GAPDH to assess the gel loading. The immunoblot is representative of four independent experiments. **e** Densities of PKCβII bands were evaluated and data are expressed as a percentage of total PKCβII (mean ± SD, n = 4). *p < 0.05 versus I/R 1 h

As was mentioned above, the I/R-induced increase in immunoreactivity of PKCβII in mitochondria was observed mainly in the CA2-4, DG regions of the hippocampus. This increase was prevented by treatment with isozyme-selective PKCβII inhibitor (βIIV5-3) what was proved by means of western blot (Fig. 3a, b) and immunogold electron microscopy (Fig. 3c). In the control, gold particles (representing bound PKCβII antibody) were found predominantly in the cytoplasm and a few in the mitochondria in both regions of hippocampus. However, mitochondrial PKCβII levels were higher following I/R injury but mainly in CA2-4, DG. The I/R-induced increase in PKCβII levels was attenuated by βIIV5-3 peptide. There was a complete absence of staining when mitochondria were incubated with anti-mouse IgG conjugated to immunogold, without prior incubation with the PKCβII antibody, excluding non-specific binding.

To show that the observed I/R-induced increase in PKCβII immunoreactivity is due to its translocation to mitochondria, a western blot analysis was carried out on the soluble and particulate (mitochondria-enriched) fractions. As Fig. 3d, e show, 5 min of ischemia and 1h of reperfusion led to a considerable transfer of the PKCβII pool from the cytosol to mitochondria. This translocation was prevented by treatment with βIIV5-3. On this basis, we can conclude that the observed I/R-induced increase in PKCβII immunoreactivity in mitochondria is the result of translocation from the cytoplasm. This result therefore entitled us to treat the βIIV5-3 inhibitor as a convenient tool confirming the hypothesis holding that PKCβII mediates a protective signaling mechanism in the ischemia-resistant region of hippocampus.

### Effect of βIIV5-3 Peptide on Neuronal Death

**Fig. 4:**
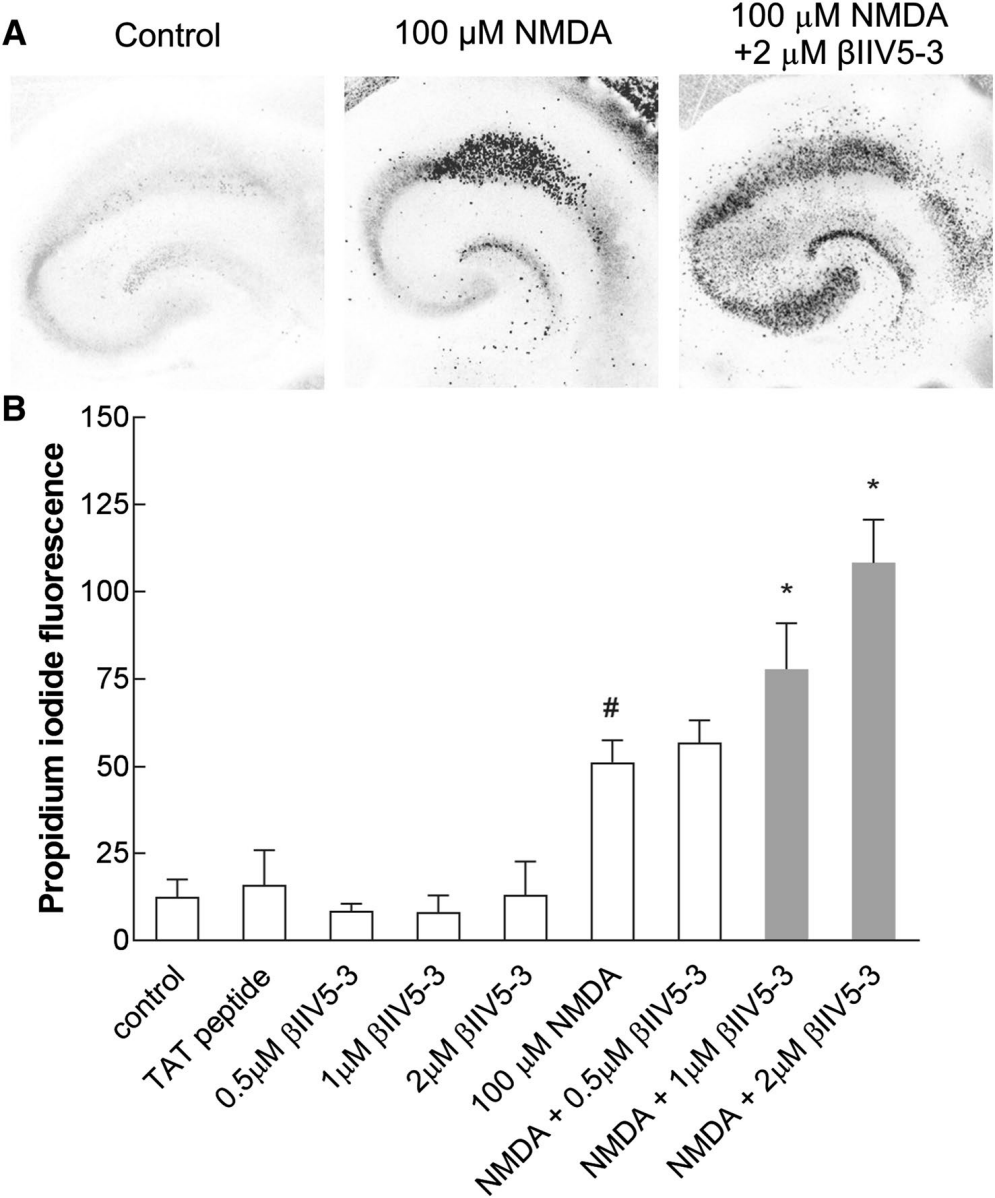
βIIV5-3 peptide-enhanced damage initially induced by NMDA in both the CA1 and CA2-4, DG regions of the hippocampus in an in vitro model of ischemia—organotypic hippocampal culture. **a** Colour-inverted fluorescent images of propidium iodide-stained hippocampal slices 24 h after treatment with 100 μM NMDA alone or together with 2 µM βIIV5-3. **b** Cell death was measured by reference to propidium iodide fluorescent intensity. The results are expressed as the mean ± SD of cells from three independent experiments. *p < 0.01 versus NMDA, ^#^p < 0.01 versus control

To determine whether selective inhibition of PKCβII could augment I/R injury, we applied βIIV5-3 in two models of ischemia, i.e. excitotoxic injury in organotypic hippocampal slices and transient cerebral ischemia in the gerbil model. The organotypic hippocampal slices were exposed to NMDA (100 µM), alone or with added (0.5, 1, 2 µM) βIIV5-3 peptide, prior to cell death being assessed 24 h after treatment. As Fig. 4a, shows NMDA treatment evoked death of pyramidal neurons in the CA1 region of hippocampal slices, while addition of the inhibitor resulted in extension of damage to the CA2-4, DG regions as well. NMDA treatment caused 51% (±6.3) cell damage expressed as a percentage of maximum fluorescence. In contrast, the simultaneous application of NMDA with 1 or 2 µM of βIIV5-3 was associated with elevated levels of damage—at 78% (±13.2) and 108% (±12.4) respectively. The 0.5 µM concentration of PKCβII inhibitor did not influence the cell damage initially induced by NMDA (57% ± 6.3). Treatment with the TAT carrier or βIIV5-3 peptide alone had no effect on injury (Fig. 4b).

**Fig. 5:**
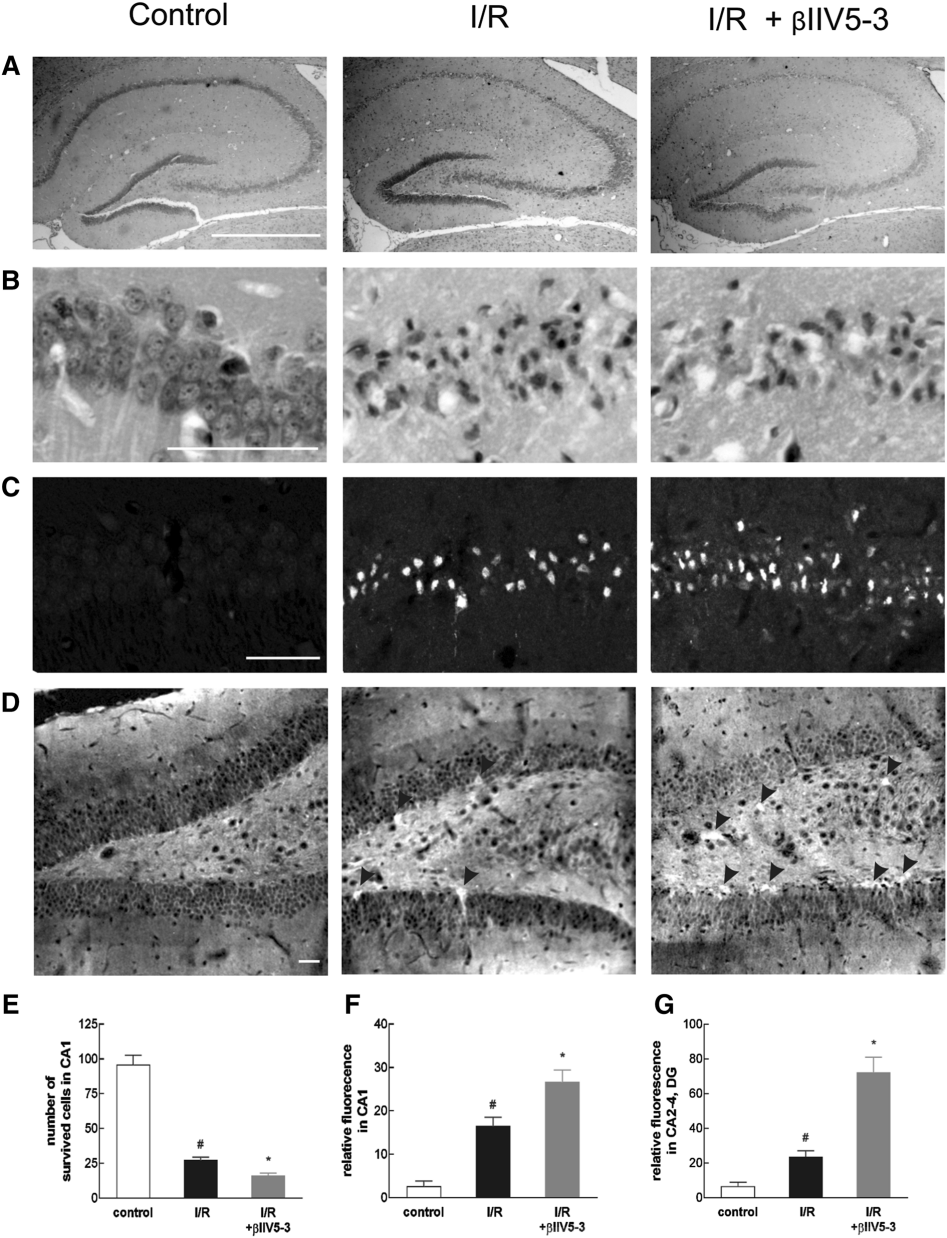
βIIV5-3 peptide-enhanced neuronal cell death in the CA1 and CA2-4, DG region after transient cerebral ischemia in gerbil model. Single dose of βIIV5-3 or saline were applied during ischemic procedure to the left carotid artery. Seven days after ischemia/reperfusion (I/R) animals were perfused with ice-cold 4% paraformaldehyde in PBS. Dissected brains were used for histological examinations on paraffin-embedded sections stained with hematoxylin and eosin (H/E) (**a, b**) and analyzed for apoptotic cell death using the TUNEL reaction (**c, d**). H/E staining on coronal sections show whole hippocampus (**a**) and enlarged area of pyramidal cells in CA1 region (**b**). The extent of cell damage of CA1 hippocampal regions were quantified as the mean number of remaining intact neurons (**e**). TUNEL staining in CA1 (**c**) and CA2-4, DG (**d**) regions of the hippocampus. The *arrowhead* indicates TUNEL-positive cells in the dentate gyrus. Fluorescent intensities of TUNEL-positive cells in the two regions of hippocampi were calculated (**f, g**). The results are expressed as the mean ± SD (n = 3). *p < 0.01 versus NMDA, ^#^p < 0.05 versus control. *Scale bar* 50 µm

Similar results were obtained in the gerbil model of brain ischemia. Transient bilateral ligation of the common carotid arteries produces specific damage to pyramidal neurons in the CA1 region (Fig. 5a–c). In control animals, the mean number of morphologically intact neurons (hematoxylin and eosin stain) in the CA1 region, counted 7 days after the sham operation, was 96 ± 6.7 (mean ± SD, n = 3). In contrast, following the ischemic episode, only ~28% (27 ± 2.2, n = 3) of neurons remained intact. The βIIV5-3 injection contributed to increases damage, with only 16% (16 ± 1, n = 3) of the CA1 hippocampal neurons surviving this insult (Fig. 5e). Hematoxylin and eosin staining showed no significant differences in damage in the CA2-4, DG regions, between the βIIV5-3-treated and ischemic groups (data not shown), though those differences were already seen in TUNEL staining (Fig. 5d). Detection of apoptotic cells confirmed that βIIV5-3 injection was associated with a significantly greater number of TUNEL-positive cells as compared with the ischemic group in the CA1 region of the hippocampus, the figures being 16.4 ± 2.1 and 27 ± 2.8, respectively (Fig. 5c, f). Furthermore, βIIV5-3 treatment was associated with relative fluorescence of TUNEL-positive cells in CA2-4, DG at a level three times greater than with the ischemic group (Fig. 5d, g). Within this area, little fluorescence of TUNEL-positive cells was observed in the control group, while the ischemic group had a fluorescence intensity that was greater than in the control. In contrast, βIIV5-3 treatment was associated with significantly more TUNEL-positive cells, which were found to be scattered in large numbers throughout the dentate gyrus (Fig. 5d, g). A greater number of TUNEL-positive cells of the dentate gyrus were labeled in the infrapyramidal than in the suprapyramidal blade. This indicates that βIIV5-3 treatment augmented I/R injury in both the CA1 and CA2-4, DG regions of the hippocampus, in both the in vitro and in vivo models of ischemia.

## Discussion

A well-known animal model for studying the pathophysiology of I/R injury is the gerbil, in which brain insult is induced by a 5-min bilateral ligation of the common carotid arteries. In this model, cerebral ischemia results in selective death of pyramidal cells in the hippocampal CA1 region, while the adjacent CA2-4, DG region remains relatively resistant. Using this model we have shown previously that I/R injury induces a translocation of PKCβ to mitochondria albeit mainly in the ischemia-resistant part of the hippocampus, in a manner that suggests the involvement of PKCβ in the ischemic resistance of that region [17].

Herein, we show that, of the two PKCβs isozymes, it is rather PKCβII than βI which appears in elevated levels in mitochondria. Mitochondria-localized PKCβII retains enzymatic activity and its elevated levels reflect PKCβII translocation from the cytoplasm to the mitochondria in response to I/R injury. This translocation is prevented efficiently if an isozyme-selective PKCβII inhibitor is applied. Moreover, inhibition of PKCβII translocation results in an increase in neuronal death after I/R injury in both in vitro and in vivo models of ischemia. In both models, βIIV5-3 treatment resulted in the spread of cell death throughout the CA1 and CA2-4, DG areas. We therefore suggest that the observed translocation may be responsible for resistance of CA2-4, DG to I/R injury, allowing PKCβII to be regarded as an important mediator of a protective signaling mechanism in the ischemia-resistant region of the hippocampus.

This is in line with other studies reporting a neuroprotective role for PKCβ in the face of ischemic injury in the central nervous system. A protective involvement was suggested in cerebral hypoxic preconditioning in mice [16, 28–30] and an oxygen-glucose deprivation model [31]. Moreover, PKCβs together with PKCα mediated phorbol ester and estrogen neuroprotection of a cultured-neuron challenge to β-amyloid peptide toxicity [32].

Other data indicate that PKCβI activation represents a key factor in protecting the integrity of neural elements in the cochlea [33]. The PKCβI isoform was found to be expressed exclusively in neural elements of the cochlea, with PKC activators such as phorbol esters and bryostatin 1 rescuing spiral ganglion neurons from cell death, and enhancing neuritic outgrowth in vitro via activation of PKCβI. There are also reports suggesting a role for PKCβI in the mechanism of EGF-mediated protection of the tight junction from acetaldehyde-induced insult [34]. Conversely, a harmful effect of PKCβ against ischemia/reperfusion has been reported in lungs, myocardium, liver and intestines [35–38].

Our data indicate that from cellular PKCβII pool only this mitochondria-localized is involved in the ischemic resistance of CA2-4, DG. This is consistent with other studies showing the potency of various PKCβII functions associated with subcellular locations [39, 40]. For example, in breast cancer the association between compartmental localization, cancer prognosis and response to therapy was observed [39]. Other studies reveal that PKCβII and PKCδ counter-regulate coronary endothelial barrier properties by targeting distinctive subcellular sites [40].

However, it is unclear what role activated PKCβs play in the neuroprotection events following I/R exposure. We speculate that translocated PKCβII regulates mitochondrial function, directing mitochondria along a pro-survival pathway. It seems likely that PKCβII interacts with mitochondrial substrates and influences activity, with the result that an anti-apoptotic mitochondrial function is induced. This suggestion is supported by our current data showing that PKCβII translocated to mitochondria retains its enzymatic activity, while inhibition of its translocation results in greater cell death in CA2-4, DG. Moreover, our previous studies identified mitochondrial proteins that can bind directly with PKCβI [17], among them (a) components of mitochondrial redox carriers forming the electron transport chain and including ATP synthase, and (b) adenine nucleotide translocase and mitochondrial creatine kinase involved in modulating the opening of mPTP through the regulation of the structure and activity of F_1_F_0_ ATP synthase [41, 42].

These results suggest that via interaction with mitochondrial proteins, PKCβ may play a role in protecting cells from damage and death [17]. The participation of mitochondrial proteins in PKCβ-mediated neuroprotection has been reported previously [16]. ATP synthase subunit δ and Ndufv2 (NADH:Ubiquinone Oxidoreductase Core Subunit V2) were identified as PKCβ-interacting proteins exhibiting significant changes following hypoxic preconditioning [16]. On the other hand, a PKCβII interaction with mitochondrial substrate was identified as an element responsible for cell death [43]. It was shown that the regulation of mitochondrial aconitase by PKCβII-dependent phosphorylation in diabetic rat heart may influence the activity of the tricarboxylic acid cycle and contribute to impaired mitochondrial function [43].

In the work described here, we used an isozyme-selective PKCβII inhibitor in the form of a six-amino-acid peptide derived from the interaction site between PKCβII and its RACK1. The efficiency and selectivity of the βIIV5-3 peptide have been demonstrated [21], and verified in a rat model of heart failure [24, 25]. In our studies, application of βIIV5-3 peptide offered effective inhibition of PKCβII translocation to the mitochondria, which in turn resulted in elevated levels of neuronal death in both an in vitro and an in vivo model of brain ischemia. Notwithstanding earlier published data [17], the application of βIIV5-3 peptide is highly justified, as the previous study used the ATP-competitive inhibitor binding to the ATP site of the kinase catalytic domain of PKCβII. The catalytic domains among PKC isozymes show a high degree of sequence homology (70%) and structural similarity, while the ATP-binding site in all protein kinases is also highly conserved. These facts may have a huge impact on inhibitor selectivity, and lead to a situation in which PKC inhibitors also affect other protein kinases, making them less suitable for experimental studies. Unfortunately then, it was the only inhibitor of what we had.

Having βIIV5-3 peptide and the expected results from in vitro studies, we decided to verify its effect in an in vivo model as well. It is worth emphasizing that the application of βIIV5-3 peptide leads to exacerbation of injury, not only in the ischemia-vulnerable region of the hippocampus, but also in the ischemia-resistant one. This result enhances our hypothesis regarding the protective potential of PKCβII.

Combining findings together, we suggest that mitochondria-localized PKCβII is involved in the ischemic resistance of CA2-4, DG. We further speculate that its protective properties are due to a number of interactions with mitochondrial proteins, which in turn guarantee PKCβII participation in the regulation of mitochondrial integrity. However, the exact mechanisms by which PKCβII mediates protective signaling remains to be elucidated.
